# Patient and provider perspectives on self-administered electronic substance use and mental health screening in HIV primary care

**DOI:** 10.1186/s13722-022-00293-7

**Published:** 2022-02-09

**Authors:** Alexandra N. Lea, Andrea Altschuler, Amy S. Leibowitz, Tory Levine-Hall, Jennifer McNeely, Michael J. Silverberg, Derek D. Satre

**Affiliations:** 1 Division of Research, Kaiser Permanente Northern California, 2000 Broadway, Oakland, CA 94612 USA; 2 Department of Population Health, Section on Tobacco, Alcohol, and Drug Use, New York University Grossman School of Medicine, 180 Madison Ave., New York, NY 10016 USA; 3 Department of Psychiatry and Behavioral Sciences, Weill Institute for Neurosciences, University of California, San Francisco, 401 Parnassus Avenue, Box 0984, San Francisco, CA 94143 USA

**Keywords:** Screening, Smoking, Alcohol, Depression, Anxiety, HIV

## Abstract

**Background:**

Substance use disorders, depression and anxiety disproportionately affect people with HIV (PWH) and lead to increased morbidity and mortality. Routine screening can help address these problems but is underutilized. This study sought to describe patient and provider perspectives on the acceptability and usefulness of systematic electronic, self-administered screening for tobacco, alcohol, other substance use, and mental health symptoms among patients in HIV primary care.

**Methods:**

Screening used validated instruments delivered pre-appointment by both secure messaging and clinic-based tablets, with results integrated into the electronic health record (EHR). Qualitative analysis of semi-structured interviews with 9 HIV primary care providers and 12 patients in the 3 largest HIV primary care clinics in the Kaiser Permanente Northern California health system who participated in a clinical trial evaluating computerized screening and behavioral interventions was conducted. Interviews were audio-recorded and transcribed. A thematic approach was utilized for coding and analysis of interview data using a combination of deductive and inductive methods.

**Results:**

Four key themes were identified: (1) perceived clinical benefit of systematic, electronic screening and EHR integration for providers and patients; (2) usefulness of having multiple methods of questionnaire completion; (3) importance of the patient–provider relationship to facilitate completion and accurate reporting; and (4) barriers, include privacy and confidentiality concerns about reporting sensitive information, particularly about substance use, and potential burden from repeated screenings.

**Conclusions:**

Findings suggest that electronic, self-administered substance use and mental health screening is acceptable to patients and may have clinical utility to providers. While offering different methods of screening completion can capture a wider range of patients, a strong patient–provider relationship is a key factor in overcoming barriers and ensuring accurate patient responses. Further investigation into facilitators, barriers, and utility of electronic screening for PWH and other high-priority patient populations is indicated.

*Trial registration* ClinicalTrials.gov, NCT03217058. Registered 13 July 2017, https://clinicaltrials.gov/ct2/show/NCT03217058

**Supplementary Information:**

The online version contains supplementary material available at 10.1186/s13722-022-00293-7.

## Background

People with HIV (PWH) have higher rates of mental health and substance use disorders (SUD) than the general population, and these conditions are associated with poorer HIV outcomes and increased mortality [1]. SUDs, including alcohol, opioid and stimulant use disorders [2–5], and depression are 2–4 times more prevalent in PWH compared to people without HIV [6, 7]. Anxiety is also common but has been under investigated relative to depression [8, 9].

Routine screening is essential for identifying these comorbidities [10–13], but is often underutilized due to lack of resources, time constraints, and stigma [14, 15]. When screening does occur, there is variability in question content, frequency, and documentation by providers [16, 17], and patients often underreport symptoms, particularly alcohol and other drug use problems [15, 18].

Patient self-report can be influenced by a variety of factors, including mode of question administration and sensitivity of content [18, 21]. Compared with face-to-face interviews, self-administered, computerized measures can facilitate more accurate reporting of stigmatized behavior [19–21], improve fidelity [14, 22, 23] and increase patient comfort [24]. On the other hand, stigma, perceived judgment, negative experiences with providers or confidentiality breaches can be barriers to disclosure of anxiety and depression [25–28] as well as substance use [29]. A trusting relationship between patient and provider can be an important facilitator to disclosure in a primary care setting and help address these barriers [23, 30, 31].

The current study aimed to investigate these issues in the context of a large screening and intervention trial focused on self-administered, computerized substance use and mental health screening in HIV primary care. This paper reports on the qualitative findings from interviews with primary care providers and patients to identify common perspectives on screening practices, as well as facilitators and barriers to accurate patient reporting in an HIV primary care setting.

## Methods

The Promoting Access to Care Engagement (PACE) trial examines a novel approach to screening, which combined substance use and mental health measures in a single, self-report questionnaire for PWH systematically administered every 6 months, coinciding with primary care appointments [32]. The PACE study was designed to evaluate the implementation, effectiveness, and cost of routine, electronic screening and brief behavioral treatments for SUDs, depression and anxiety. The qualitative data reported here focused on screening rather than subsequent interventions delivered by behavioral health specialists. The study occurred in the 3 largest HIV primary care clinics (Oakland, Sacramento, and San Francisco) in Kaiser Permanente Northern California (KPNC), which collectively serve over 5000 PWH. Patients were asked to complete 2 validated, self-administered screening measures prior to a scheduled appointment: The Tobacco, Alcohol, Prescription medication, and other Substance use (TAPS) instrument [33], which has been shown to have good sensitivity and specificity for identifying problem use in the general primary care population, particularly for tobacco, alcohol, and marijuana; and the Adult Outcomes Questionnaire (AOQ), which includes the Patient Health Questionnaire (PHQ-9) for depression [34] and the Generalized Anxiety Disorder (GAD-2) for anxiety [35], both of which have also been shown to have high levels of sensitivity and specificity in the same population. These were combined in a single questionnaire (the TAPS/AOQ).

KPNC offers patients an online portal that allows access to appointments, lab results, other health care information and secure messaging with their providers. Patients with access to the portal were invited via secure message to complete the TAPS/AOQ online prior to their visit, and those who did not were invited to complete it on a tablet upon arrival in the clinic. TAPS/AOQ responses were automatically recorded in the patients’ electronic health record (EHR) and were visible to providers and behavioral health specialists embedded in HIV primary care (Sacramento) or general primary care (Oakland and San Francisco). Key clinical trial outcomes include screening completion rates; utilization of specialty addiction and mental health treatment; and HIV viral control based on EHR data [32]. Outcome analyses are in process.

The focus of analysis here are qualitative telephone interviews conducted with providers and patients from participating clinics to better understand barriers and facilitators associated with implementation and clinical utility of substance use and mental health screening. Verbal informed consent and permission for recording and transcription were obtained. Participants in the provider interviews did not receive compensation. Participants in the patient interviews received a $50 gift card after the interview. Study procedures were approved by the KPNC and University of California, San Francisco Institutional Review Boards.

### Participants

Interviewees were providers and adult patients from each of the three participating clinics. Patient interviewees who previously completed the TAPS/AOQ were selected via convenience sampling by participating clinicians. The study team partnered with providers to identify participants for recruitment from all 3 facilities with varying degrees of self-reported substance use and mental health symptoms. Eligible patients received a study information sheet from their provider either at their in-person clinic visit or via secure message after a virtual visit. Interested patients allowed their provider to give their contact information to study staff, who completed the telephone interview. We provide descriptive characteristics of participants, including demographics (age, sex, race, and HIV risk group) and scores on the TAPS, GAD-2, and PHQ-9, and compare these to the overall sample of PACE patients from all three study clinics.

### Interview guides

Provider interviews included questions about substance use and mental health screening practices pre-trial; experience with the TAPS/AOQ, including facilitators and barriers to implementation; perceived success of implementation; feedback from patients regarding the screening; and value and feasibility of utilizing the TAPS/AOQ as part of clinic operations after study conclusion. See Additional file 1: Appendix S1 for the provider interview guide. Patient interviews included questions about experiences with substance use and mental health screening prior to TAPS/AOQ implementation; experiences completing the questionnaire; facilitators and barriers to completion; and any interactions with providers based on screening results. See Additional file 2: Appendix S2 for the patient interview guide.

### Data collection

Provider and patient interviews were conducted by two investigators (DDS and MJS) and a Masters level team member (ANL). Clinician interviews lasted 30–45 min and patient interviews lasted up to 30 min over a 9-month period. Interviews were audio recorded and transcribed verbatim. Interviews were conducted until study investigators believed thematic saturation was reached [36, 37] based on the concepts of data adequacy [38–40] and data source triangulation [41].

### Qualitative analysis

We used a thematic approach, combining deductive and inductive reasoning, for coding and analysis [42, 43]. This approach was chosen to identify and evaluate both explicit and implicit perspectives provided by interviewees. With input from the research team, author AA developed separate codebooks for the provider and patient interviews based on interview guides and field notes. Four authors (AA, DDS, ANL and ASL) independently coded a quarter of the transcripts in each category. Differences in coding were resolved via consensus, and final codebooks were established. Data analysis was managed using NVivo statistical software version 12 (QSR International) and followed standard methods for qualitative research to ensure analysis was systematic and verifiable [44, 45].

## Results

Nine providers and 12 patients participated in interviews from all three sites. Providers (8 physicians and one nurse practitioner) included 6 men and 3 women. Interview participant characteristics and those of patients in the overall PACE cohort are included in Table 1. The interview and intervention samples had similar demographics (age [median = 59 vs. 55], gender [92% male vs. 92% male], risk group [83% MSM vs. 76% MSM], and race/ethnicity [50% White; 17% Black; 33% Hispanic vs. 53% White; 18% Black; 16% Hispanic]). Findings show that while demographic characteristics and HIV risk group were similar in the interview sample, interviewees scored higher on measures indicative of substance use disorder risk, anxiety and depression.

**Table 1:** Characteristics of patient interview cohort vs. overall PACE cohort

Total patients	Pt. interview cohort		PACE cohort	
	12	100%	2892	100%
Substance use (high/med.)				
Any risk	10	83%	1759	61%
Depression/anxiety				
PHQ9 (≥ 10)	4	33%	381	13%
GAD2 (≥ 3)	3	25%	378	13%
Demographics				
Age, median (IQR)	59	(41–61)	55	(46–62)
Sex, *n* (%)				
Male	11	92%	2656	92%
Female	1	8%	236	8%
Risk group, *n* (%)				
MSM	10	83%	2189	76%
Heterosexual	2	17%	346	12%
IV drug use	0	0%	187	6%
Other	0	0%	170	6%
Race, *n* (%)				
White	6	50%	1532	53%
Black	2	17%	535	18%
Hispanic	4	33%	474	16%
Other	0	0%	351	12%

Key themes identified from patient and provider interviews are described below. Additional illustrative quotes, by theme, are shown in Table 2.

**Table 2:** Additional patient and provider quotes on TAPS/AOQ screening

Theme	Providers	Patients
Perceived clinical benefits	“It’s definitely helpful for me, because I [received] that data before I even saw the patient. So then I can really focus on…the behavior…the treatment plan, and discussing the next steps….”	“I know it's part of them providing holistic care around everything about my health, including HIV, so I'm glad they ask the questions. I hope more doctors do that with their patients 'cause I know that doctors are rushed and they just have to get through things sometimes”
	“It’s helped making our practices more consistent, because sometimes patients don’t present as if they have mental health issues or substance use issues. So routinizing the questions and applying them equally to all patients is helpful”	
Value of multiple modes of administration	“I think having both modes of collection was helpful, because it captured from different patient populations…as much as it was possible to do things before the visit, I think that was very helpful for workflow”	
Patient–provider relationship	“I had a few patients who reacted pretty negatively … it certainly was somewhat of a limitation in a subset of patients who…were put off by the nature of the questions particularly around substance abuse. After I had a conversation with them about it, things were always fine, but…that conversation could’ve maybe happened ahead of time to alert patients that things like this were coming and why”	“[I'm] still an old-school guy. I’m way more apt to speak in person than doing something online, pretty much across the board”
Barriers to completion	“[A patient] raised the concern about documentation of substance use in this way in their chart and it affecting their disability and benefits in a negative way. They were worried that disclosing substance use would cause a private disability insurance to kick them off”	“I had a little bit of hesitancy, which I have almost every time I do one of those [AOQ] questionnaires… Sometimes you feel like if you answer it, they’ll lock you up or there will be some big response to it. So, you think twice about it when you answer it”
	“Some of our patients tend to be far more protective of their confidentiality than patients who are HIV-negative or who are not used to the stigma that’s associated with HIV. And so a lot of them are not active on [the patient portal] or are very nervous about their medical information leaking”	“The main reason why privacy is a concern is because everything that you do…it’s bound by HIPAA, but you never know if it could get in the wrong hands or like if the nurse that knows you can access that information or something like that”

### Theme 1: Clinical benefits and value of systematic screening

Patients reported that prior to implementation of the TAPS/AOQ during the trial, substance use screening focused mainly on tobacco and alcohol. Mental health screening was “more vague or ambiguous, like ‘how are you doing?’; ‘how's everything going?’” All patients supported the new screening questions, with one patient indicating “not only is it [the] physical aspect [of HIV] that you’re dealing with, but it’s also an emotional and mental thing because it’s a really big lifestyle change…It’s good to know where a person’s mental health lies, and then, shortly after, it became like a substance abuse thing…So, I think it’s appropriate to ask those types of questions.”

Providers reported that the TAPS/AOQ reinforced the importance of screening and increased their awareness of the prevalence of substance use and mental health issues for PWH. Most providers also felt that the TAPS/AOQ identified more patients with these issues, particularly those that were less severe, and whose conditions they were previously unaware of. One provider noted, “The nice thing with [the TAPS/AOQ] is it certainly has picked up things that I haven’t asked about, and I can think of people that throw in… party drugs, ecstasy, that I may not have picked up on or they may not have told me. But it comes out in those questionnaires.”

Providers reported different practices regarding frequency of mental health and substance use screening before the PACE trial, often dependent on whether a given patient was known to have existing problems. Providers differed in where they would document such issues in the EHR. Only tobacco and alcohol screening were regularly completed in a uniform manner [46, 47]. Although providers utilized some validated instruments such as the PHQ-9 prior to TAPS/AOQ implementation, mental health screening practices varied by clinician. Providers found that consistent screening practices and standardized documentation of results helped inform clinical decisions. One said, “I would share results with the patients in terms of their TAPS/AOQ responses and we could follow them together to look for improvements or areas of lack of improvement and perhaps worsening over time.”

Inclusion of screening for substances other than alcohol and tobacco meant that providers gained more information about their patients’ use, while self-administration preserved limited appointment time for other patient needs. One noted: “It’s really helpful, because…it saves appointment time in order to have that information there….You’re walking into a situation where you’ve done that screen, you know how much time you’re going to prioritize to that.”

### Theme 2: Value of different screening modalities

All patients reported it was easy to complete the TAPS/AOQ, regardless of self-administration method (patient portal or in-clinic tablet). They also reported preferring different methods to access the TAPS/AOQ depending on how and when they access care. Some were familiar with the portal and regularly utilized it to interact with providers, and therefore preferred the convenience of online completion, while others stated that they were more likely to complete the questionnaire in the clinic, when they felt they were in a “patient mindset.” Some indicated that tablet completion helped them transition from hectic daily routines to focusing on their healthcare upon arrival in the clinic. One patient described the difference as: “I did it on my computer, and that takes like pre-preparation in order to actually pre-access it. And, once I'm actually present within the [clinic] building itself, I guess it's more so trying to be present and prepare myself for my doctor’s appointment.”

Providers felt that having online and tablet options for TAPS/AOQ completion captured different patient populations and both were valuable. Online completions were convenient for patients who preferred to complete the questionnaires in advance and eased the administrative burden on clinic workflows. Tablet completions were valuable for those who lacked internet access or were not registered to use the portal: “We certainly don’t want to miss those folks, because in some ways they may be even a higher risk patient population.”

### Theme 3: Impact of patient–provider communication and relationship on screening

The quality of patient–provider communication and relationships were important factors in patients’ level of comfort disclosing substance use and mental health issues. Patients appreciated when providers mentioned their TAPS/AOQ results during their visit, whether there were issues or not, and said such discussions made it more likely for them to disclose issues in the future. One patient commented about the value of their provider having this information: “[The provider mentioning TAPS/AOQ results] makes me feel good because then it feels like he’s actually paying attention to what I put time on…. I felt like he cared about what I was going through, and that made me feel good.”

Patients also reported that developing a relationship with their provider over time and discussing questions and responses in person made them more likely to report substance use and mental health issues. One stated: “I want that personal connection…. I feel fortunate having these long-term relationships with the doctors that I can say anything and feel comfortable saying anything that I want.” Patients and providers both reported that availability of TAPS/AOQ screening results informed patient–provider discussions and proved an important opportunity to strengthen and improve the relationship. Even for patients who chose not to complete the screening, receipt of the questionnaire could still prompt a conversation regarding why the questions were being asked and/or why it was not completed.

Both patients and providers discussed two key aspects of the secure message: (1) sent from the patient’s provider, versus from a general health system email address, and (2) included an explanation regarding the importance of screening completion. One provider stated: “I think people, when they get emails, you know, ‘from your doctor,’ they think it really comes from your doctor, that your doctor sits down and writes this and sends it to you, without recognizing that 90 percent of those, your doctor doesn’t even know get sent. So, they’re like, ‘Wow. Why are you asking me this?’ Not like, ‘Why is [the health system] doing this?’ It was very personal.” A patient noted: “I'm glad that it was explained to me what it was all about and why I was being asked those questions, and once I was told, it was like, ‘Oh, okay. No problem. I understand that now.’”.

### Theme 4: Barriers to completion—privacy/confidentiality and questionnaire fatigue

The most frequently mentioned barriers to TAPS/AOQ completion were concerns regarding privacy and confidentiality. Patients were concerned that their answers might be used in a negative or discriminatory manner related to their care, employment or benefits. One patient stated: “I mean, everything is a part of your medical record. So, I feel that you always kind of have to be wary of how in depth you go with everything.” Some patients were also concerned with the sensitivity of the TAPS/AOQ content and their responses being misunderstood because the questionnaire does not capture the nuances of substance use, recovery, and mental health. For example, several reported having to answer affirmatively to TAPS/AOQ questions due to the questionnaire’s wording, but not having the opportunity to contextualize their answers (e.g., previous substance use and mental health issues resolved many years prior).

Providers also reported that, while many PWH were familiar with substance use and mental health screening, some had concerns regarding confidentiality, and preferred that providers document substance use and mental health responses in their clinical notes rather than as answers to screening tools. They felt that patient privacy was more protected in a free text clinical note versus structured EHR data that is more readily retrievable, including by health plan administrators, other clinicians and staff. One provider noted: “Some patients have had issues with disclosure around substance use. And primarily they will be untruthful on the questionnaire because it doesn’t come from me directly. And they will say, ‘I actually use, X, Y, Z, but I didn’t report it here,’ and primarily for confidentiality and employment reasons, they don’t want that in their medical record—even if it gets noted in my note, it’s different than if they just kind of answered on the questionnaire.”

Finally, another common barrier to completion was a sense of burden or fatigue due to past receipt of other health surveys. The KPNC healthcare system regularly utilizes online questionnaires in many departments for patients who use the patient portal. Patients sometimes reported feeling overwhelmed by the number of questionnaires they were asked to complete, which could lead to them not completing any of those requested. Furthermore, many providers expressed concern about patients being asked to complete the TAPS/AOQ on a regular basis, especially in cases where there was an established patient–provider relationship and no previous history of symptoms. For example, one stated: “[One patient] really [took] offense, and that was basically conveying… ‘You know me. Why would you ask me these questions? You know I don’t smoke. You know I don’t drink. You know I have never used drugs.’ The same provider stated: “I am concerned that patients that score very low on all of these measures, when queried again in 6 months, might get some fatigue around it and feel frustrated.”

## Discussion

This qualitative study examined patient and provider perspectives on the implementation of systematic, self-administered substance use and mental health screening in three large HIV primary care clinics in an integrated health care system. We identified 4 themes among patients and providers regarding their experiences utilizing the TAPS/AOQ tool: (1) perceived clinical benefits; (2) value of multiple modes of administration; (3) importance of the patient–provider relationship as a facilitator to substance use or mental health disclosure; and (4) privacy/confidentiality concerns and questionnaire fatigue as primary barriers to screening completion.

Our findings suggest that, although substance use and mental health screening was occurring prior to TAPS/AOQ implementation, screening practices were highly variable, which is consistent with previous reports of substance use and mental health screening in similar settings [16, 17]. As noted above, standard KPNC practice already routinely screened all primary care patients for alcohol and tobacco use [46, 47], but the TAPS/AOQ enabled screening for tobacco, alcohol, and all major drug classes in a single instrument. Given the recently revised US Preventive Services Task Force recommendation that routine drug screening should be part of high-quality primary care for all adults [48], this aspect of the TAPS/AOQ makes it well suited to meet provider needs, particularly in HIV primary care. Providers reported the benefit of consistent screening practices for all substances, standardized documentation of results, and periodic rescreening and that TAPS/AOQ implementation increased their awareness of patients’ substance use and mental health problems, enabled them to track risk levels and symptoms over time, and often enhanced patient/provider relationships. Of note, providers found value in the long-term monitoring of screening results for patients who initially reported symptoms, both for clinical decision making and strengthening the patient–provider relationship through informed discussions. Although the AOQ depression and anxiety measures have been validated for monitoring over time, the TAPS and other substance use screening tools have not.

All patients indicated that the self-administered screening was highly acceptable, consistent with previous findings [33, 49]. Prior research has provided evidence for patient acceptability of a self-administered TAPS format [33, 49, 50], while our results build on those findings to show the utility of multiple modes of self-administration (online patient portal and in-clinic tablet) in meeting the needs of different patient populations. Both patients and providers felt that having both options available allowed for more flexibility in administration and an increased likelihood of completion. This flexibility may be especially important to capture younger populations who regularly utilize technology to access care, as well as safety net populations who may not have internet or smartphone access.

Previous research has also indicated that a good provider-patient relationship, while important in all medical care, is particularly valuable for patients with substance use and mental health concerns, where having established trust with a provider can reduce feelings of shame around disclosure [23, 30, 31, 51]. Our results further showed that the most important facilitator for completion of electronic screening was a strong provider-patient relationship in which feelings of trust have been established. Patients and providers felt that discussing TAPS/AOQ responses led to strengthened relationships by increasing feelings of trust and patient comfort with provider, which led to more accurate responses from patients. For those who completed the TAPS/AOQ online, receiving it directly from their provider and including an explanation of its importance were key factors in their decision to complete the screening.

Finally, PWH may place greater weight on confidentiality and privacy [25–28], which is reflected in reports that the sensitivity of drug use and mental health information was a common barrier. Some patients reported hesitation to complete the screening because they worried that their responses, if formally recorded in their EHR, might negatively impact their care, benefits, or employment; consistent with previous findings [30, 52]. Although patients were not formally asked if perceived benefits of screening outweighed risks, the overall positive comments suggest that they thought screening was valuable and, despite the inclusion of sensitive topics, content areas were relevant to their care. A strong patient–provider relationship characterized by open discussion and lack of judgment, in which the provider can explain and emphasize the importance of capturing these results on an ongoing basis, can allay these fears and increase the likelihood of accurate self-report. Patients may also suffer from questionnaire fatigue over time, potentially impacting satisfaction with care and decreasing the quality of the screening [53–57]. Careful consideration of administration schedule is warranted, including mechanisms to vary frequency based on patterns of patient responses, e.g., fewer screenings for those who consistently report minimal substance use or mental health symptoms.

### Study limitations

There were several limitations to this study. A small sample was interviewed, patient participants were primarily male (although this is representative of the population of PWH in KPNC), and the study was based in a private health care system. Convenience sampling by providers for patient interviews may have resulted in limiting participants to those who had a more positive experience with the clinic or the study in general. Additionally, although demographic characteristics of the interview sample were very similar to that of the overall PACE cohort, interviewees scored higher on measures indicative of substance use disorder risk, anxiety and depression. Interviewee perspectives may be less representative of the clinic population, which includes patients with lower or no risk. However, given the intent of the study to evaluate screening and treatment for these conditions, we believe that perspectives of people with higher-severity problems were important to include. Given the barriers noted, such as privacy concerns and questionnaire fatigue, future implementation efforts with screening systems such as this must carefully address these challenges. For example, assessing responses after multiple administrations could provide insight on how regular screening may specifically influence responses and relationships with providers over time, particularly for those who report few or no substance use problems or mental health symptoms.

## Conclusions

This study evaluated patient and primary care provider perspectives regarding newly implemented self-reported substance use and mental health screening practices in HIV primary care. The use of the self-administered electronic screening tool proved successful in providing systematic screening and documentation of results. Both modalities of completion (online vs. tablet) as well as the tool itself had high levels of patient acceptability, and self-administration both preserved appointment time for providers to address patient care needs as well as captured substance use and mental health issues not previously reported. Regardless of mode of self-administration, a strong provider-patient relationship appears to be the most important factor in screening completion and accuracy, helping to mitigate patient privacy concerns. Regular screening and review of results between providers and patients can serve as an opportunity to strengthen this relationship and improve substance use and mental health interventions in primary care.

## Supplementary Information

**Additional file 1: Appendix S1.** Provider Interview Guide. Qualitative interview guide used to complete interviews with providers.

**Additional file 2: Appendix S2.** Patient Interview Guide. Qualitative interview guide used to complete interviews with patients.

## Data Availability

The qualitative data analyzed is not publicly available to protect participant identities. Anonymized copies of interview transcripts are available from the corresponding author on reasonable request. Copies of the study interview guides as well as the screening instrument are available upon request.

## Abbreviations

AOQ: Adult Outcomes Questionnaire
EHR: Electronic health record
GAD-2: Generalized Anxiety Disorder
KPNC: Kaiser Permanente Northern California
PACE: Promoting Access to Care Engagement
PHQ-9: Patient Health Questionnaire
PWH: People with HIV
SUD: Substance use disorder
TAPS: The Tobacco, Alcohol, Prescription medication and other Substance use Instrument
