# Supplementary material for: Patient and provider perspectives on self-administered electronic substance use and mental health screening in HIV primary care
Source: Addict Sci Clin Pract. 2022 Feb 9;17:10. doi: 10.1186/s13722-022-00293-7 (PMC8827178; doi:10.1186/s13722-022-00293-7)
Supplement: Supplementary file 1 — Additional file 1: Appendix S1. Provider Interview Guide. Qualitative interview guide used to complete interviews with providers. [file 13722_2022_293_MOESM1_ESM.docx]

**APPENDIX S1 – PROVIDER INTERVIEW GUIDE**

**PACE: Semi-structured Interview for Key Informants**

**Interviewees: HIV primary care providers (PCPs), case managers, nurse practitioners**

***To get us started, I’d like to learn about your role in the clinic and on the PACE project.***

**Intro/warm-up questions:**

1. To start with, please tell me what your position is and a little about your current role in the clinic.
2. Prior to the start of the PACE project, to what extent were you involved in screening and/or treatment for for substance use and mental health problems?
   1. How did you typically screen patients?
   2. How did you provide treatment (or referrals) if needed?

***Now I’d like to ask you about the implementation of* self-administered SUD and mental health screening integrated into the medical record, *which is a key feature of the PACE Project. I’m especially interested in how screening may have impacted patient care.***

**Overall pre-visit screening questionnaires:**

1. To what extent do you think that rollout of the TAPS-AOQ was successful – i.e., that the screening process is consistently followed, and results meaningfully utilized by clinicians and staff? [clinic]
   1. What factors helped with implementation?
      1. E.g., staffing levels, clinician characteristics such as comfort with technology, member population characteristics, and other clinic resources and priorities
   2. Which factors do you think posed challenges for implementation?
2. To what extent have the TAPS-AOQ screening procedures and results helped make you aware of patients’ mental health symptoms and/or substance use issues in ways that you weren’t previously? [patient/intervention]
   1. How is using the TAPS-AOQ different from how you used to screen for these problems, either formally or informally?
   2. Have PACE screening results changed anything about your practice of discussing substance use or mental health with your patients?
   3. How confident are you in raising substance use and mental health screening results with patients?
   4. Has this changed at all, as a result of TAPS-AOQ screening, and/or having a BHS working in the clinic?
   5. How helpful have you found the screening results in motivating behavior change and/or referring patients to services (either to the BHS in-clinic or to specialty care)?
      1. In what ways do you use the screening results to inform and motivate your patients?
      2. In what ways do you use screening results in communicating with other members of the primary care team?
3. When do you typically review patients’ TAPS-AOQ responses? For instance, is it usually before, during, or after an appointment - or not at all? What, if anything, gets in the way of consistently reviewing patient responses? [intervention]
4. Which tool(s) do you use to view patient TAPS-AOQ responses – e.g., HealthConnect flowsheet, HC smartlink that pulls responses into your appointment note, iHIV, DOR staff message regarding PHQ item 9?
   1. Which methods are most clinically useful and why? [intervention]
   2. Which methods did you NOT find useful and why? [intervention]
5. What kind of feedback, both positive and negative, have you had from patients about the screening?
6. If you can, please comment on any pros and cons of the three screening modalities available in the clinic (secure messaging, tablet, and suspended hyperspace) in terms of your preferences, clinic work flow, ease of use generally, patient acceptability/use, etc. [intervention]

1. The study team at the Kaiser Division of Research is supporting the clinic by tracking when patients are due to receive a TAPS/AOQ (every 6 months, and when they have an upcoming in-person appointment), and attaching the questionnaire to that appointment, which is what triggers the flag for reception to give the patient a Tablet at registration. DOR is also communicating directly with patients to invite them to complete the questionnaire prior to the appointment via KP.org.
   1. Once this support ends, would you want TAPS/AOQ administration to be sustained, e.g., do you see it as valuable? [intervention]
   2. What ideas do you have regarding how routine computerized screening can be sustained? [intervention]

**BHS care delivery questions (for PCSs and other non-BHS clinicians):**

1. The second major component of the PACE Project includes training and support of a behavioral health specialist (BHS) in the clinic. Can you comment on the extent to which having a primary care-based behavioral health specialist has been valuable (or not valuable) in addressing mental health or substance use problems? [intervention]
   1. How often do you refer patients to a BHS?
   2. To what extent has your use of BHS services changed as a result of the PACE project?
2. What do you think has supported BHS integration in your clinic? [intervention/clinic]
   1. Has TAPS/AOQ screening been useful in supporting BHS integration?
   2. How have team meetings or other direct communication with BHSs (e.g., by phone or email) been useful?
3. Can you comment on any challenges that may have negatively impacted effective BHS-delivered care in the clinic? [clinic]
   1. E.g., warm hand off vs. scheduled appointments, BHS availability, BHS training or skillset, patient expectations about what a BHS is able to provide.
4. What do you think would help to sustain or improve BHS integration in the clinic, in the future? [clinic]
   1. E.g., support from leadership in the medical center, communication with psychiatry/addiction medicine, communication in the clinic such as team meetings, or EHR-based communication.
5. Is there anything else we haven’t talked about that would give us a better understanding of how the clinic and/or KPNC can improve and sustain mental health and substance use screening and treatment integration in primary care, especially among patients with HIV?
