# Supplementary material for: Patient and provider perspectives on self-administered electronic substance use and mental health screening in HIV primary care
Source: Addict Sci Clin Pract. 2022 Feb 9;17:10. doi: 10.1186/s13722-022-00293-7 (PMC8827178; doi:10.1186/s13722-022-00293-7)
Supplement: Supplementary file 2 — Additional file 2: Appendix S2. Patient Interview Guide. Qualitative interview guide used to complete interviews with patients. [file 13722_2022_293_MOESM2_ESM.docx]

**APPENDIX S2 – PATIENT INTERVIEW GUIDE**

**PACE: Semi-structured Interview for Key Informants**

**Interviewees: HIV patients who had a TAPS/AOQ screening questionnaire attached to an appointment with a participating PACE provider.**

Thank you so much for taking the time to speak with us today. I’m going to start by asking some questions about how you receive your HIV care at Kaiser Permanente and your relationship with your HIV care physician, as well as some questions about any substance use or mental health screening you may have received before this project began.

**Intro/warm-up questions:**

1. To start with, please tell me a little bit about your care in the clinic.
   1. How long have you been receiving your HIV care at the KP [Oakland/Sacramento/San Francisco] medical center?
   2. How and how often do you typically interact with them (e.g. secure messaging in KP.org, telephone visits, video visits, in person visits, etc. on a monthly, annual, as needed basis)?
   3. How satisfied are you with how substance use and mental health issues are addressed within your HIV care clinic?
2. Sometime in the past year, you may remember receiving the **Tobacco, Alcohol, Prescription medications and other Substances Tool/Adult Outcomes Questionnaire (TAPS/AOQ**) questionnaire, which asked you questions about your tobacco, alcohol prescription medication and other substance use as well as your mental health. This was a questionnaire that you filled out yourself, either online before your visit or on a tablet when you checked in for your appointment. Before this electronic questionnaire was available, your provider may have asked you about these topics during your regular visits with them.
   1. If you recall discussing these topics with your provider prior to receiving the TAPS/AOQ questionnaire, what kinds of questions did they ask you? Was this something you discussed on a regular basis?
   2. How did you feel about discussing this information with them?
   3. Prior to the past year, did you ever receive any kind of follow up from your HIV provider or from any other clinical provider after your visit about any of these topics?
      1. If yes, were you satisfied with the follow up you received?
      2. If no, would you have liked to have received follow up?

Now I’d like to ask you about your experience completing the questionnaire that this project is focused on, called the **TAPS/AOQ**. We will ask about your experience receiving the questionnaire and anything that made it easier or harder for you to complete it. We’re especially interested in hearing about ways we could improve the delivery of this questionnaire in the future.

**For patients who are active on KP.org:**

1. Do you remember receiving an email message through KP.org asking you to complete the questionnaire?
   1. **If yes,** how easy or how difficult was it to access this email and the questionnaire online? What problems did you encounter when trying to access the message or questionnaire?
   2. What device(s) were you using to access the message (e.g. smartphone, desktop, tablet)? How easy or how difficult was it to read and navigate through the pages?
      1. On a scale of 1 to 10, with 1 being the hardest and 10 being the easiest, how hard was it for you to complete the questionnaire on your device?
2. Did you complete the questionnaire online before your appointment? Why or why not?

**For patients who are NOT active on KP.org or did not complete the questionnaire online prior to their appointment:**

1. Did you receive a tablet and instructions from the reception staff regarding filling out the TAPS/AOQ questionnaire when you checked in to your appointment?
2. Did you complete the questionnaire on a tablet at your appointment?
   1. What factors affected your ability to complete the questionnaire on the tablet?
   2. How easy or how difficult was it to use the tablet? How easy or how difficult was it to read and navigate through the pages?
   3. Did you have enough time to complete the questionnaire before you saw your doctor?

**STOP HERE for patients who did not complete the questionnaire, and skip to CONCLUSION section**

**For patients who DID complete the TAPS/AOQ questionnaire:**

Now I’d like to ask you about your experience answering the questions on the **TAPS/AOQ**. This questionnaire covers potentially sensitive topics that you may or may not feel comfortable answering and/or discussing with your provider.

1. The TAPS/AOQ asks about substance use and mental health symptoms. Did you feel like it was appropriate for your provider to ask you about this? Why or why not?
2. Did you think the questionnaire asked too much or too little about these topics? Please tell me what makes you think or feel that way?
3. Did you have any concerns about privacy when answering the questionnaire? Any other concerns? If so, why?
4. Did your provider mention your answers during your visit? Did you feel like their response to your answers was appropriate? Why or why not?

**STOP HERE for patients who were not referred to BHS care after completing the questionnaire, and skip to CONCLUSION section**

**For patients who scored high in one or more categories on the TAPS/AOQ questionnaire:**

Finally, I’d like to ask about your experience with any clinical follow up follow up with a behavioral health provider while you were in the clinic or soon afterward?

1. Did your provider say that they would like you to meet with a behavioral health specialist (BHS) regarding clinical care?
   1. Did they introduce you to the other provider in person or did they let you know that you would be receiving a follow up call?
   2. Did they discuss what options this person would be able to provide?
2. Did you speak with a BHS after your visit to the clinic?
   1. **If no**,
      1. Would you have liked to have received follow up from a BHS clinician? Why or why not?
      2. What would make it easier for you to connect with a BHS in the future?
   2. **If yes**,
      1. How long after your visit with your primary care provider did you meet with the BHS?
      2. Did you or the BHS mention or reference any of your TAPS/AOQ answers at ANY time?
      3. Did you find the BHS follow up useful? Why or why not?
      4. Were you satisfied with the mental health and substance use care options that the BHS was able to provide for you? Why or why not?
      5. What could have improved your experience with the BHS?

**CONCLUSION**

1. Is there anything that I did not ask about regarding your experience completing the TAPS/AOQ questionnaire and any care that you may have received based on your responses that you would like to add?

In order to send you your $50 incentive for participating, I will need to verify your mailing address. Could you please confirm where I should send your gift card?

Name: ____________________________

Address: __________________________

City, State, Zip: _____________________

Thank you again for taking the time to speak with us today, we appreciate your participation and feedback on the project! If you have any questions or think of any additional information you would like to add after this interview, please feel free to contact me at [DOR STAFF PHONE NUMBER] or [DOR STAFF EMAIL ADDRESS].
